# Exploring irradiated granular flows with rapid heating for concentrated solar thermal energy collection and storage

**DOI:** 10.1016/j.isci.2025.112164

**Published:** 2025-03-04

**Authors:** Shin Young Jeong, Devesh Ranjan, Zhuomin M. Zhang, Peter G. Loutzenhiser

**Affiliations:** 1 George W. Woodruff School of Mechanical Engineering, Georgia Institute of Technology, Atlanta, GA 30332-0405, USA

**Keywords:** Physics, Applied sciences, Engineering

## Abstract

Gravity-driven granular flows along an inclined plane were considered with exposure to different levels of irradiation. Experiments were performed with average normal radiative heat fluxes of 400, 500, and 600 kW/m^2^, and the spatial and temporal free-surface velocity and temperature fields were measured. Granular flow behavior changed under different irradiation, primarily due to an increase in particle friction changing with increased temperatures. A transient, pseudo-two-dimensional heat and mass transfer model was developed to capture the relevant heat transfer mechanisms in these flows. The model considered irradiation penetration, effective conduction heat transfer, and non-uniform velocity profiles. The predicted steady state free-surface temperatures were well-correlated to experimental measurements with Pearson’s correlation coefficients >0.9. The model predicted a large temperature gradient in the flow due to irradiation attenuation, flow velocity variations in depth, and low effective thermal conductivity. This study effectively captured the heat transport of the irradiated inclined plane granular flows.

## Highlights

• Granular flow behaviors on inclined plane explored under non-uniform radiative heating
• Significant changes in flow behavior observed at elevated temperatures
• A pseudo heat transfer model adopting discrete element method was developed
• A steep temperature gradient along the flow depth was predicted

## Graphical abstract

**Figure:**
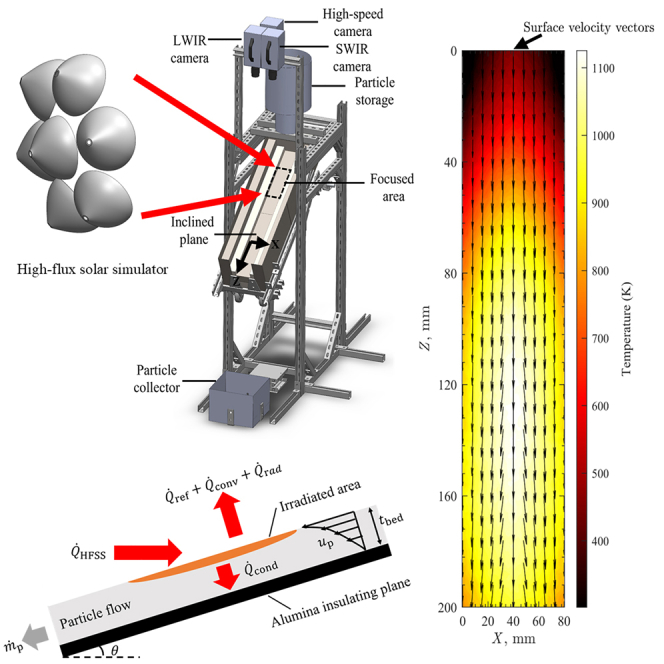

## Introduction

Concentrated solar power (CSP) technology affords a renewable pathway toward electricity production that avoids the environmental impacts associated with fossil fuels.^1^^,^^2^^,^^3^ Continuous operation outside of diurnal periods is enabled when coupling CSP with thermal energy storage (TES).^4^^,^^5^ Among the different possible pathways for the next-generation CSP technology, particle-based thermal energy storage is a promising route that has stimulated widespread interest.^6^^,^^7^^,^^8^ Inexpensive ceramic and silica-based solid particles are used as heat transfer and TES media, which are heated on-sun in solar receivers with the potential to reach temperatures exceeding 1,300 K.^9^ Moreover, coupling high temperature particle receivers to high-efficiency power cycles (e.g., supercritical carbon dioxide [sCO_2_] Brayton power cycle) has the potential to improve the solar-to-electric efficiency.^10^^,^^11^

Sintered bauxite particles have been investigated in directly irradiated solar receivers due to favorable thermal energy transport and storage attributes, including high temperature resilience and high thermal capacity, which enables heat retention over extended period.^12^ Carbobead particles composed of Al_2_O_3_, SiO_2_, Fe_2_O_3_, and TiO_2_ with a high solar absorptance >90% are excellent candidates for the particulate TES media in the next generation CSP linked with TES.^13^^,^^14^^,^^15^ These sintered bauxite particles were utilized in direct heating solar receivers in various granular flow form, including in free-fall,^6^ along inclined planes,^16^^,^^17^ through obstructed flows,^7^^,^^18^ and integrated into centrifugal rotating receivers.^8^^,^^19^ Solar receivers utilizing inclined plane granular have higher efficiencies due to the dense granular flows effectively absorbing and transporting concentrated solar irradiation, thus, both experimental and simulation studies were conducted to understand relevant heat transfer mechanisms and assess the performance.^16^^,^^17^^,^^20^^,^^21^ Omitted from these studies were investigations of granular flow behavior changes due to temperature, which potentially impacts the thermal efficiency of the CSP system and requires a different design paradigm for optimal particle resident time.

The inclined solar particle receiver offers three principal advantages: (1) it combines heat collection and storage, making it suitable for integration with dish power generation systems; (2) the closed design minimizes particle losses during circulation; and (3) particle velocity is controlled to achieve a higher outlet temperature.^22^ An inclined particle receiver was developed in other work with ZnO particles forming a moving bed that slides down the incline under gravity, reaching internal temperatures of up to 1,435 K.^23^^,^^24^ A similar design was later introduced with quartz glass windows to reduce heat and particle loss and a pneumatic control valve to regulate particle residence time.^25^ Experimental results demonstrated an outlet temperature of ∼938 K with a particle flow rate of ∼7.5 g/s and incident radiative power of ∼8 kW_th_. A simple heat and mass balance analysis indicated that reducing the flow rate to ∼5 g/s potentially increases the outlet temperature and efficiency to ∼1,205 K and 71%, respectively. Inclined flows have been further studied in solar thermal chemical reactors, including designs with dense particle-gas flows for energy storage via two-step thermochemical cycles.^16^^,^^20^ This reactor configuration included an inclined granular flow within a horizontal cavity and a quartz window for vacuum operation and direct solar irradiation.^20^ Initial modeling predicted thermochemical performance for Co_3_O_4_ reduction, using empirical correlations to track granular flow and coupling them with radiative and particle energy models to optimize reactant conversion and prevent overheating. Later studies focused on Al-doped calcium manganite for different operating temperatures and higher energy density.^16^ Experiments showed that flow behavior was impacted by particle residence time, bed depth, and inclination angle, which significantly affected reactor performance, with higher angles improving efficiency by reducing conductive resistance. However, recent studies have suggested that in-depth analysis of flow physics and associated heat transfer in inclined flows for receiver/reactor applications remains limited, highlighting a need for further research.

Recent concerted efforts to numerically model the inclined granular flows at elevated temperatures^26^ have incorporated measured temperature-dependent flow properties of the particles.^27^^,^^28^ These flow properties encompass particle shape and size distributions, elastic properties, coefficient of restitution, and coefficients of friction. A significant temperature dependence for granular flows has led to alterations in particle residence times and mass fluxes, primarily attributed to changes in particle frictional properties. Experimentation in parallel with numerical modeling on the inclined granular flows was conducted with electrically heated particles, reaching temperatures up to 1,300 K.^29^ A marked reduction in free-surface velocities and mass flow rates was observed due to changing flow properties. The variation in particle flow properties with particle temperatures is of paramount importance for heat and mass transfer modeling in granular flows. Despite these advancements, a significant gap remains in research concerning changes in granular flow behaviors within actual solar receivers, particularly when the flowing particles are directly heated by the high-flux radiation, which usually produce non-uniform temperature distributions within the flow.

Significant efforts have focused on developing numerical heat and mass transfer models to predict performance of various types of particle-based solar receiver/reactors, including free-falling,^7^^,^^30^^,^^31^ obstructed flow,^25^^,^^32^^,^^33^ centrifugal,^19^ moving-bed,^34^^,^^35^ and fluidized-bed.^36^ Most studies of CSP systems with particle flow do not consider particle flow behavior changes with temperature in solar receivers.^37^^,^^38^ This oversight can be particularly significant for solar receivers utilizing inclined granular flows, where variations in flow behavior, such as changes in flow velocity and bed thickness, can have a profound impact on overall system performance.^39^ In these systems, alterations in flow velocity can lead to deviations from expected particle temperatures due to changes in exposure time to concentrated solar irradiation. Faster flow rates may reduce the exposure time, preventing particles from reaching the desired temperature, while slower flow rates potentially lead to overheating or inefficient energy use. Moreover, changes in particle flow thickness, especially the formation of thicker flows due to particle accumulation, can cause substantial temperature variations in the outlet particle temperature. In thicker particle beds, particles located in the inner layers are more difficult to effectively heat up. This uneven heating can result in significant temperature gradients within the particle bed, affecting the thermal performance and efficiency of the solar receiver. These variations in particle flow behavior are critical because they directly impact the thermal transport processes within the receiver. Efficient thermal transport is essential for maximizing the efficiency of energy conversion in CSP systems. When particle flow behavior is not fully understood and optimized, it can lead to a decrease in the overall efficiency of the solar receiver, causing deviations in the performance estimation of large MW_th_ to GW_th_-scale CSP systems. A thorough understanding and consideration of particle flow behavior changes with temperature are essential for accurate system design and performance analysis of CSP systems. Addressing these factors can lead to more reliable performance estimations and enhanced efficiency in large-scale solar thermal energy applications.

The aim of this work was to comprehensively analyze granular flows of sintered bauxite particles along an inclined plane directly exposed to non-uniform radiative heat flux from a high-flux solar simulator (HFSS). This study provides the first experimental observations of changes in the granular flows due to temperature-dependent frictional properties as the flow is rapidly heated by high radiative heat fluxes. The results were used to further illicit the intricate relationship between temperature variations, granular flows, and heat transfer dynamics. A series of flow experiments along an inclined plane was performed at average normal radiative heat fluxes between 400 and 600 kW/m^2^ with the bed height, free-surface velocities, steady-state mass flow rates, and free surface particle temperature measured. A numerical solution was developed for modeling heat and mass transfer of granular flow exposed to high radiative heat flux. The model was formulated using a two-dimensional, one-phase continuum approach, and measured free-surface velocities were introduced to provide particle velocity distribution in the flows. Comparisons were made between predicted and measured free-surface particles temperatures. The results were further used to investigate the heat transfer within granular flows influenced by a non-uniform particle velocity at different depths of the bed. The findings are important for optimizing and evaluating particle-based solar receiver performance and assessing the impact on granular flow variations due flow properties that change with temperature.

## Results

### High flux radiation alters granular flow behaviors on an inclined plane

**Figure 1:**
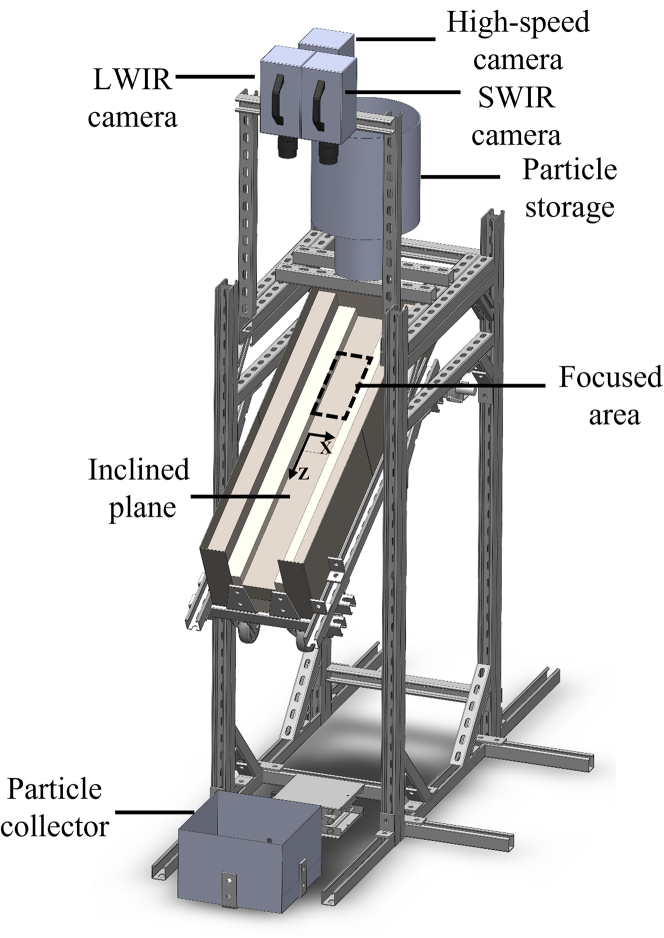
Fabricated inclined flow setup for HFSS

**Figure 2:**
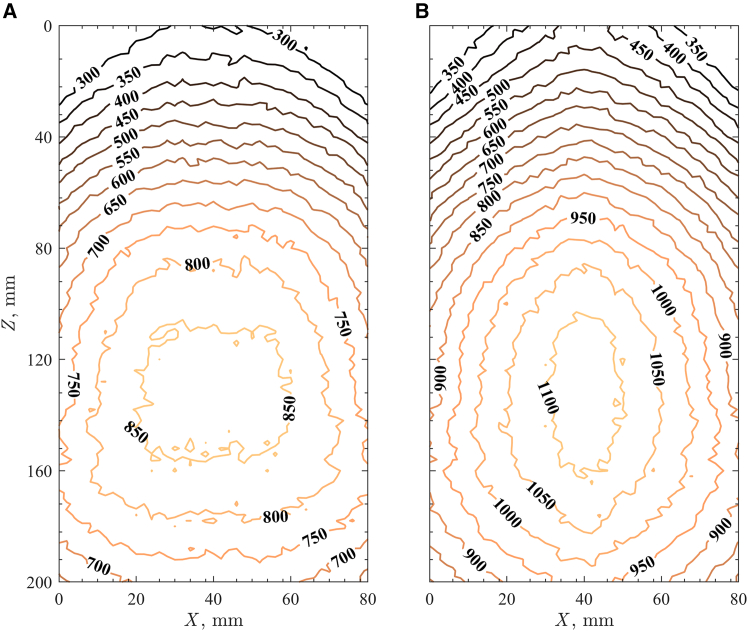
Contour of free-surface temperatures in K of sintered bauxite granular flow on the inclined plane exposed to average normal radiative heat fluxes (A) 400 kW/m^2^. (B) 600 kW/m^2^.

The impact of non-uniform radiative heat flux on an inclined granular flow was examined in a HFSS.^40^ An inclined flow experimental apparatus was fabricated, and the inclined plane was positioned in the HFSS focal plane (Figure 1). The inclined plane was fabricated from high-density rigid alumina board at angle of 27° from horizontal. The experimental region dimensions were a length of *L* = 200 mm and a width of *W* = 80 mm. Sintered bauxite particles (Carbobead CP 30/60, CARBO) with effective diameters of *d*_p_ = 418 ± 59 μm^24^ were introduced along the inclined plane. Particle free-surface temperatures at quasi-steady state were measured at three different average normal radiative heat fluxes of $\bar{q}$″ _HFSS_ = 400, 500, and 600 kW/m^2^, and temperature contour maps are given in Figure 2 for (1) $\bar{q}$″_HFSS_ = 400 kW/m^2^ and (2) $\bar{q}$″_HFSS_ = 600 kW/m^2^. The time to reach steady state for the granular flows was <3 min. The particle free-surface temperature changes with time of *∂T*_p_/*∂t* for the granular flow initially were very high for ≤ 1 min, indicative of rapid heating, after which the *∂T*_p_/*∂t* decreased significantly until reaching quasi-steady state. The maximum *T*_p_ of the granular flow was slightly below the center of the lamp focus by 30 mm due to advection in the granular flow. An increase in the particle free-surface temperature in the flow direction of *∂T*_p_/*∂Z* was observed due to increased lamp power toward lamp focus. Maximum *T*_p_ = 1,043 K for $\bar{q}$″_HFSS_ = 400 kW/m^2^ and 1,168 K for $\bar{q}$″_HFSS_ = 600 kW/m^2^ were observed at *Z* = 130 mm. The granular flows above *Z* = 130 mm experienced temperature drops due to decreases in the spatial lamp power and strong radiative heat losses to the surroundings. Temperature contour maps for $\bar{q}$″_HFSS_ = 600 kW/m^2^ showed larger *T*_p_ differences in *Z* and *X* directions of the flow compared to $\bar{q}$″_HFSS_ = 400 kW/m^2^.

**Figure 3:**
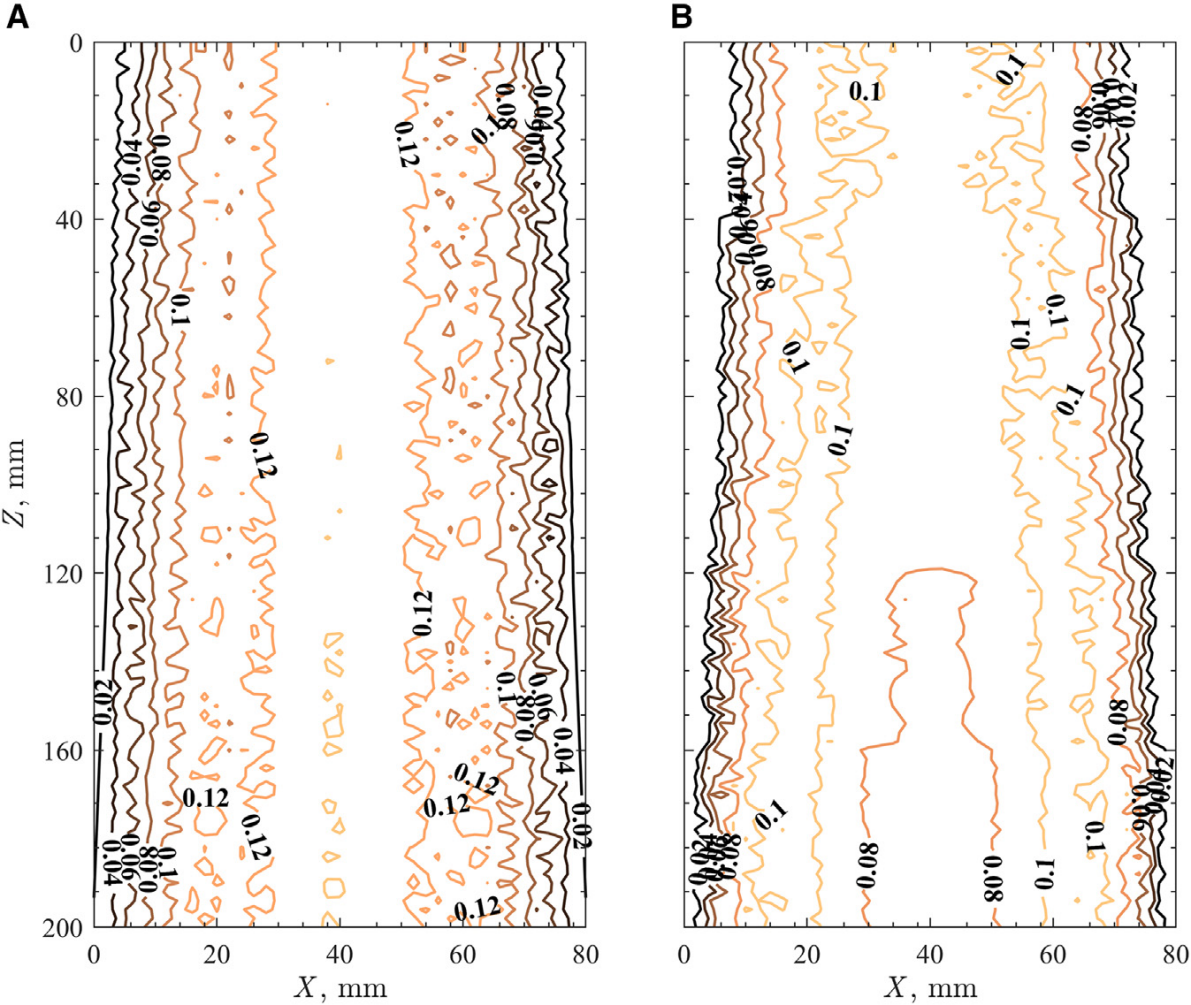
Contour of free-surface velocities in m/s of sintered bauxite granular flow on the inclined plane (A) Room temperature. (B) Average normal radiative heat flux of 600 kW/m^2^.

Particle free-surface velocity fields measured and analyzed by PIV and velocity contour maps at the thermal steady state are given Figure 3 for (1) the room temperature and (2) $\bar{q}$″_HFSS_ = 600 kW/m^2^. When the granular flows along the inclined plane were not exposed to high radiative heat fluxes, the particles moved fastest at the center of the flow. Particle free-surface velocities of *u*_p,s_ > 0.12 m/s were observed within 30 < *X* < 50 mm. Closer to the side walls (*X* < 30 mm and *X* > 50 mm), the *u*_p,s_ decreased and some of the particles near the wall (*X* < 5 mm and *X* > 75 mm) were stagnant due to high frictional interactions with the alumina wall and the flowing particles. The granular flows accelerated while traveling along the inclined plane and after *Z* > 120 mm at the center regions, and *u*_p,s_ > 0.14 m/s were observed. The increase in *u*_p,s_ with *Z* resulted in fewer stagnant particles near the wall.

Significant differences in the flow behavior were observed in the flow experiment with $\bar{q}$″_HFSS_ = 600 kW/m^2^, primarily due to the large *T*_p_ variations under these conditions. The temperature strongly influenced particle friction, integral for understanding flow behavior. The non-uniform radiative flux generated a large *T*_p_ gradient within the flow, causing local variations in flow frictions and resulting in distinct flow behavior. The fastest rates of *u*_p,s_ were not observed in the central region (30 < *X* < 50 mm), however, two *u*_p,s_ peaks were observed in the region slightly shifted away from the center due to higher *T*_p_ at the central region of the flow compared to the outer region of the flow from the temperature contour map (Figure 2). The shift occurred because elevated *T*_p_ in the central region led to stronger particle-to-particle friction, due to enhanced sliding friction coefficients.^29^ The *u*_p,s_ was above 0.12 m/s for the two peaks, and the physical distance between the peaks was ∼20 mm at *Z* = 100 mm and gradually increased to 50 mm at *Z* = 200 mm due to a large increase in *T*_p_ in the central region of the flow. Within the upper section (*Z* < 100 mm) of the central region of the flow, *u*_p,s_ remained between 0.12 and 0.1 m/s, but decreases in *u*_p,s_ were observed for *Z* > 100 mm where *T*_p_ of the flow reached the maximum. Strong particle-to-particle friction in this region resulted in *u*_p,s_ below 0.1 m/s and continued to decrease until the flow exited the irradiated region. Differences in the flow behavior were also observed in the side regions of the flow where *u*_p,s_ decreased rapidly closer to the alumina walls, and more stagnant particles were observed compared to the granular flow at room temperature. The *u*_p,s_ measurement indicated strong frictional forces exerted in the central region of the flow due to elevated temperature, which redirected the particle flows toward the outer region of the plane with less friction.

**Figure 4:**
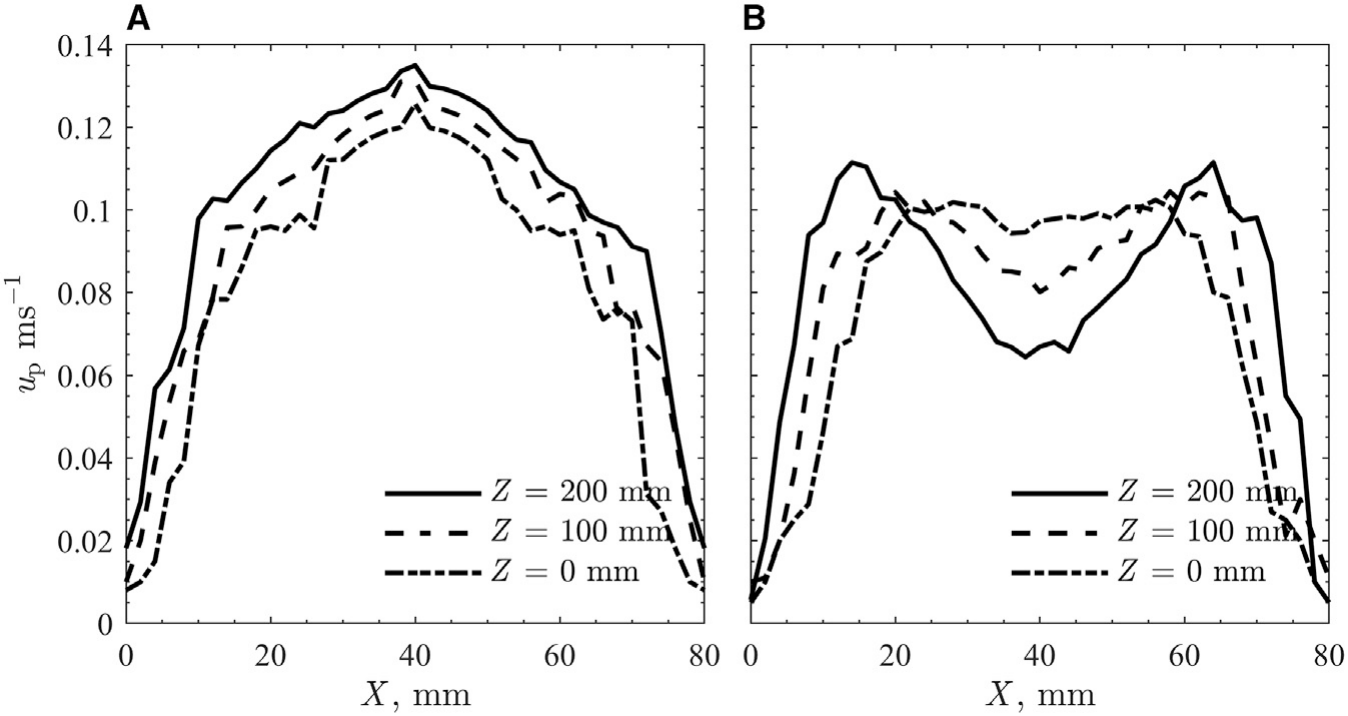
Particle free-surface velocities along the plane width at location *Z* = 200 mm (solid line), *Z* = 100 mm (dashed line), and *Z* = 0 mm (dashed-dotted line) for the inclined plane granular flow (A) Room temperature. (B) Average normal radiative heat flux of 600 kW/m^2^.

The *u*_p,s_ at different locations in the flow direction were analyzed to further understand granular flow behaviors exposed to non-uniform radiative heat flux. The *u*_p,s_ in the *X* direction at location of *Z* = 0, 100, and 200 mm are presented in Figure 4 for (1) the room temperature and (2) $\bar{q}$″_HFSS_ = 600 kW/m^2^. The granular flow along the inclined plane at room temperature showed a similar trend of *u*_p,s_ peaking at the center (*X* = 40 mm) and decreasing near the side walls for all three different *Z* locations. Increases in *u*_p,s_ were observed along the inclined plane. The granular flow exposed to $\bar{q}$″_HFSS_ = 600 kW/m^2^ showed an M-shaped *u*_p,s_ profile in *X* direction for all three *Z* locations (Figure 4B). The *u*_p,s_ was quite uniform in the central region (20 < *X* < 60 mm) with a small decrease at the center. Two *u*_p,s_ peaks appeared at *Z* = 100 mm for *X* = 20 and 60 mm with a difference of Δ*u*_p,s_ = 0.02 m/s compared to the *u*_p,s_ at the center. The two *u*_p,s_ peaks became more obvious at *Z* = 200 mm and were shifted slightly toward the side walls at *X* = 15 and 65 mm with increases in the Δ*u*_p,s_ = 0.045 m/s. The average *u*_p,s_ in *X* direction decreased with increases in *Z*, but the *u*_p,s_ peaks increased, indicative of particles tending to move toward regions with weakest frictional forces. The trend of the M-shaped in the *u*_p,s_ profile was also observed in the flow experiments with lower $\bar{q}$″_HFSS_, but the Δ*u*_p,s_ were smaller than the results from the flow experiment with $\bar{q}$″_HFSS_ = 600 kW/m^2^.

The means and standard deviations of steady state particle mass flow rates of the inclined plane granular flow were measured by a load cell integrated with the particle collector placed at the end of the inclined plane. The particle mass flow rates of $\dot{m}$ and the bed thickness of *δ*_bed_ at the room temperature and three different $\bar{q}$″_HFSS_ conditions were measured and are listed in Table 1. A significant decrease in $\dot{m}$ was observed for different $\bar{q}$″_HFSS_. The findings are well-correlated to the previous numerical and experimental results of granular flows on the inclined plane at elevated temperature.^24^^,^^26^ A lower steady state $\dot{m}$ at ∼1073 K was observed due to a sharp increase of >50% in the interparticle coefficient of static sliding friction between room temperature and 1073 K. The granular flows along the inclined plane at *T*_amb_ showed the *δ*_bed_ = 3.54 ± 0.5 mm and slight increase in *δ*_bed_ was observed with increasing $\bar{q}$″_HFSS_.

**Table 1:** Steady state particle mass flow rates from inclined plane experiments at room temperature and different average normal radiative heat flux

Conditions	$\dot{m}$*,* g/s	*δ*_bed_, mm
Room temperature (*T* = 300 K)	31.5 ± 2	3.54 ± 0.23
$\bar{q}$″_HFSS_ = 400 kW/m^2^	21.7 ± 2	3.87 ± 0.32
$\bar{q}$″_HFSS_ = 500 kW/m^2^	21.2 ± 2	3.92 ± 0.25
$\bar{q}$″_HFSS_ = 600 kW/m^2^	20.8 ± 2	3.95 ± 0.28

### Depth-dependent particle velocity influences thermal transport

**Figure 5:**
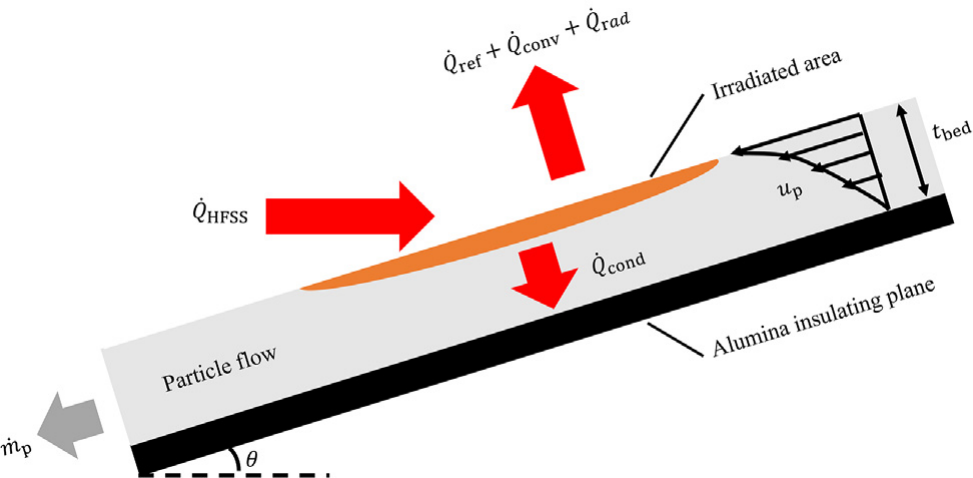
Schematic of heat and mass transport of the granular flow along the inclined plane with incident high-flux radiation

The heat and mass transfer model was exercised to better understand the heat transfer mechanisms within the granular flow when subjected to different $\bar{q}$″_HFSS_. The pseudo, two-dimensional, continuum, non-steady heat transfer model to asses the flow domain in both the flow and *δ*_bed_ directions was developed and focused on the centerline of the exposed flow (*X* = 40 mm) where the incident *q"*_HFSS_ was the strongest. The flow properties and incident radiative heat flux on the flow were assumed to be symmetric with the centerline of the flow. Thermophysical properties of bauxite particles were adopted and are listed in Table 2.^6^^,^^9^^,^^13^^,^^15^^,^^27^ The schematic of heat and mass transport in the granular flow is given in Figure 5, depicting the cross-sectional view of the granular flow on *YZ* plane. The high-flux radiation from HFSS at the heat flow rate of $\dot{Q}$_HFSS_ was directed onto the surface of the granular flow with bed thickness of *δ*_bed_. The absorbed irradiation was transferred to the lower layer of the flow and followed the Beer-Lambert law. Any unabsorbed radiation was reflected from the surface at the heat flow rate of $\dot{Q}$_ref_. The heat transfer mechanisms were convective and radiative heat transfer to surroundings repesented as $\dot{Q}$_conv_ and $\dot{Q}$_rad_, respectively. The heat from the bed surface was transferred through the bed via conduction from particle contacts, convection through air voids, and thermal radiation. The overall heat transfer was captured with an effective thermal conductivity model.^41^^,^^42^ Grid sizes of Δ*Y* = Δ*Z* = 0.5 mm were adopted that provided a good compromise between model fidelity and the computational load. The *q*"_HFSS_ projected on the inclined surface was calculated from the previously measured flux maps. The spatial and temporal *u*_p_ in both *Y* and *Z* directions adopted the measured *u*_p,s_ at *X* = 40 mm, and *u*_p_ at the inner bed was calculated from the measured *u*_p,s_ and *δ*_bed_. The average particle volume fraction of *φ* for the granular flow did not significantly change with $\bar{q}$″_HFSS_ where *φ* = 0.57 at $\bar{q}$″_HFSS_ = 600 kW/m^2^ and *φ* = 0.59 at the ambient temperature. Although *δ*_bed_ increased with temperature rise, $\dot{m}$ decreased, resulting in relatively constant *φ* at different testing conditions.

**Table 2:** Thermophysical properties of particle

Parameter	Value
Solid particle density (*ρ*_p_)	3300 kg/m^3^
Particle specific heat (*c*_p,p_)	1200 J/kg·K
Particle diameter (*d*_p_)	418 μm
Solar weighted absorptance (*A*_p_)	0.895
Particle emittance (*ε*_p_) at 973 K	0.75

**Figure 6:**
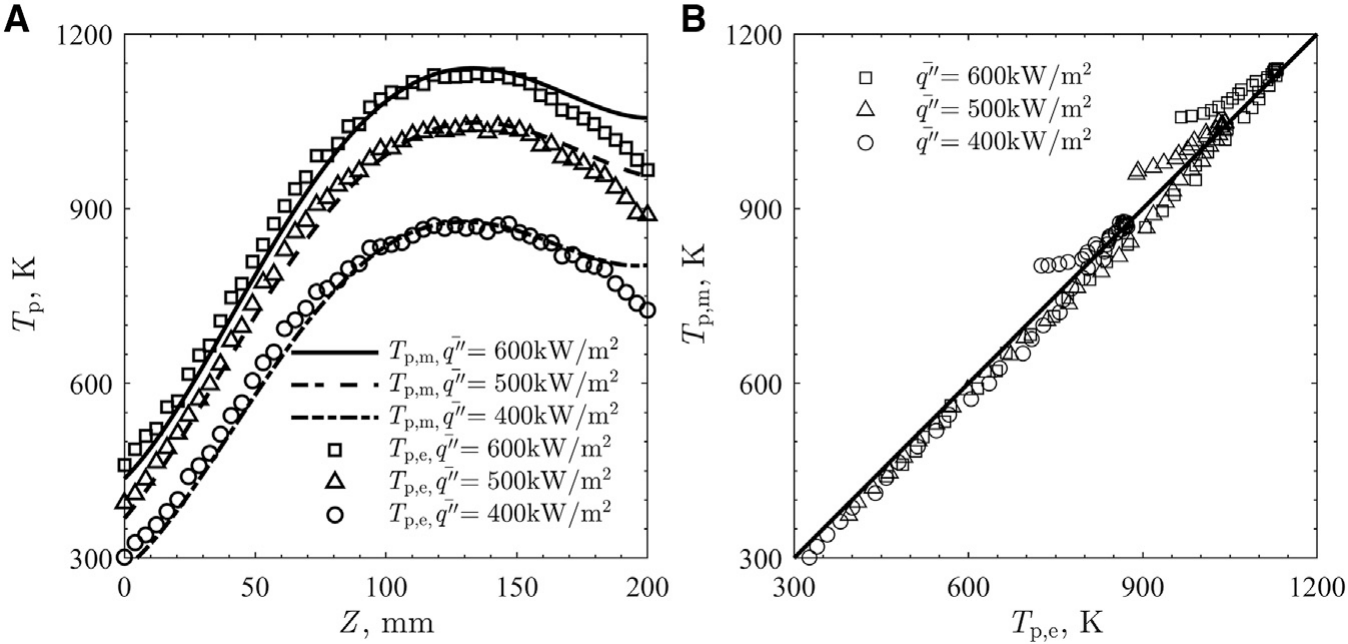
Steady state surface particle temperature of the flow at X = 40 mm (A) Results obtained from experiment (marker) and simulation (line). (B) Parity plot for comparison between experiment and simulation.

The heat transfer in the granular flow at different $\bar{q}$″_HFSS_ was modeled, and the results are given in Figure 6 for (A) steady state surface *T*_p_ obtained from the experiment and the model and (B) a parity plot comparing between the experiment and the model for different $\bar{q}$″_HFSS_. The surface *T*_p_ at the center of the flow (*X* = 40 mm) were compared between the experiment and the model. The modeled *T*_p,m_ was well-correlated with the overall trend of the experimentally obtained surface *T*_p,e_ in the flow direction (*Z* direction) where the peak surface *T*_p,e_ was observed as slightly below the center of the beam focus. However, the model exhibited a slight under-prediction of *T*_p_ at the surface prior to the peak and over-prediction following the peak. The differences between *T*_p,m_ and *T*_p,e_ were largest at the exits of the flows. The model showed that surface *T*_p_ decreased after the peak, though the rate of change was smaller compared to the experimental result. While the irradiation drops largely near the flow exit, the simulated surface *T*_p_ remained elevated due to an increase in *T*_p_ in the internal flow, driven by irradiation penetrating into the flow. The discrepancy at the exit potentially occurred as the model was not fully capturing the heat loss in the flow, including heat losses from the flow surface to the ambient and conductive heat loss to the testing structure. Pearson’s correlation coefficient between *T*_p,m_ and *T*_p,e_ were used to quantify the results for different $\bar{q}$″_HFSS_, defined as:

(Equation 1) $$r=\frac{n\left(\sum T_{p,m}T_{p,e}\right)-\left(\sum T_{p,m}\right)\left(\sum T_{p,e}\right)}{\sqrt{\left[n\sum {T_{p,m}}^{2}-{\left(\sum T_{p,m}\right)}^{2}\right]\left[n\sum {T_{p,e}}^{2}-{\left(\sum T_{p,e}\right)}^{2}\right]}}$$

where *n* is the number of pairs of the variable. *r* estimates the correlation between two variables and their relationships where -1 ≤ *r* ≤ 1, where 1 indicates perfect positive correlation, -1 indicates perfect negative correlation, and 0 indicates no correlation. The computed *r* for different $\bar{q}$″_HFSS_ is given in Table 3 and were highly correlated with *r* > 0.9. The *r* for lower $\bar{q}$″_HFSS_ showed better prediction compared to higher $\bar{q}$″_HFSS_.

**Table 3:** Pearson’s correlation coefficients for predicted and measured steady state surface particle temperature

${\bar{q}}^{\prime \prime }$_HFSS_, kW/m^2^	*r*
400	0.991
500	0.992
600	0.990
Total	0.991

**Figure 7:**
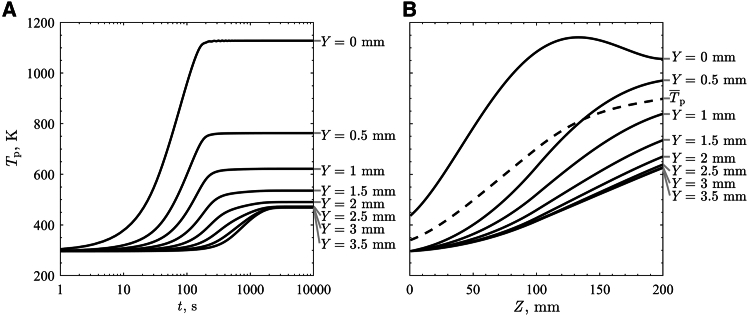
Simulated results from the developed heat and mass transfer model for an average normal radiative heat flux of 600 kW/m^2^ (A) Transient particle temperatures at different depths of the flow at the location of the max surface particle temperature (*Z* = 130 mm). (B) Steady state particle temperatures at different depths of the flow (solid line) and an average steady state particle temperature (dashed line).

Simulated *T*_p_ at different *Y* locations within the flow at $\bar{q}$″_HFSS_ = 600 kW/m^2^ were investigated with the model and results are given in Figure 7 for (A) temporal changes in *T*_p_ at the location of peak steady state surface *T*_p_ = 1148 K (*Z* = 130 mm) and (B) steady state *T*_p_ in the *Z* direction. The surface *T*_p_ reached a steady state at ∼3 min, which correlated well with the experimental results. The results showed an increase in *t* to reach the steady state with an increase in *Y*. The bottom most layer took almost 30 min to reach the steady state. A particle flow layer with faster heat up reached thermal equilibrium quicker due to the removal of heat from in the simulation domain more quickly than the layer with slower heat up. A clear difference in *T*_p_ and changes with *t* were observed for the flow at *Y* < 2 mm due to the large change in *u*_p_. Due to small changes in *u*_p_ for the flow at *Y* > 2 mm, changes in *T*_p_ with *t* followed a similar trend and reached a very similar steady state *T*_p_. Due to attenuation of irradiation in the flow, the amount of energy delivered below *Y* < 2 mm was below 2% of the total power, but due to low *u*_p_, the *T*_p_ reached 470 K. When the granular flow reached steady state, elevated *T*_p_ near the flow surface were observed. Gradual increases in *T*_p_ at the inner flow were observed with the bottom of the flow reaching *T*_p_ = 626 K at the exit. Such increases in the inner flow of *T*_p_ resulted in a small decrease in the surface *T*_p_ beyond the peak. A large difference in *T*_p_ in *Y* direction was likely due to the attenuation of irradiation in the flow and the low effective thermal conductivity of *k*_p,eff_ that impeded effective heat transfer from the hot surface to the inner layers. The faster moving upper particle layers were also transporting the absorbed heat out of the simulation domain, impeding heat transfer to the bottom particle layers. The mean bulk *T*_p_ was calculated to represent the total thermal energy transported by the granular flow, considering the variation of *u*_p_ and *T*_p_ with *Y* resulting at ${\bar{T}}_{p}$ = 798 K at the exit.

Steady state *T*_p_ results (Figure 7B) showed the surface *T*_p_ (*Y* = 0 mm) peaked at *Z* = 130 mm and decreased due to reduction in *q"*_HFSS_. *T*_p_ below the surface (*Y* > 0 mm) continuously increased throughout the simulation domain (0 ≤ *Z* ≤ 200 mm). *T*_p_ at *Y* = 0.5 mm showed a steep increase until reaching *Z* = 150 mm after which the increasing rate reduced due to a decrease in the surface *T*_p_. The results clearly showed limited heat transfer to the bottom of the flow where the *T*_p_ at the bottom layer (*Y* = 3.5 mm) reached 626 K at the exit (*Z* = 200 mm). The steady state ${\bar{T}}_{p}$ of the flow in *Z* direction was analyzed. ${\bar{T}}_{p}$ showed a continuous increasing trend in *Z* direction and the increasing rate reduced with a decrease in the surface *T*_p_. The results clearly show that ${\bar{T}}_{p}$ was impacted by the top few layers with the most thermal energy transported. Enhanced solar receiver efficiencies are potentially possible based on these results by utilizing directly heated granular flows, and reducing the *δ*_bed_ results in a potential increase in the *T*_p_ of the granular flow. The heat and mass transfer model provides unique insights into temperature distribution and heat transfer within the granular flow to potentially inform solar receiver/reactor designs.

## Discussion

An experimental study was conducted for the granular flow along the inclined plane exposed to non-uniform radiative heat fluxes from a high-flux solar simulator to inform solar particle receiver development. A series of flow experiments along an inclined plane was performed at average normal radiative heat fluxes of 400, 500, and 600 kW/m^2^ with the bed height, free-surface velocities, steady-state mass flow rates, and free surface particle temperature measured. The results showed that temperature-dependent particle friction properties, combined with heating from non-uniform heat fluxes, created localized friction variations within the flow. The average normal radiative heat flux of 600 kW/m^2^ resulted in a granular flow with the maximum elevated temperature up to 1,148 K. Significant changes in the granular flow behavior were observed when exposed to high radiative heat fluxes compared to the flow at the room temperature. While the granular flow at the room temperature showed the fastest free-surface velocity at the center of the flow, the heated granular flow showed a decrease in the free-surface velocity at the center where the peak temperatures were observed. The enhanced inter-particle friction at elevated temperatures resulted in two peak free-surface velocities away from the center leading particles to flow where the friction was lower. The particles near the edge of the plane remained stagnant due to the high frictional forces between the alumina wall and particles and more stagnant particles were observed with increasing the incident heat flux. Overall, the free-surface velocities and mass flow rates of the granular flow tended to slow down and particle flow bed heights increased slightly with an increase in the particle temperature.

A numerical heat and mass transfer model for the gravity-driven granular flow along an inclined plane directly heated by radiative heat fluxes was developed and compared to experimental measurements. A one-phase, two-dimensional, continuum approach considering depth dependent flow velocities was used to capture the relevant physics in the model. The model adopted experimentally measured steady state free-surface velocities, mass flow rates, and bed thickness. The model calculated heat diffusion within the flow adopting the effective thermal conductivity from the Zehner, Bauer, and Schlünder model. The penetration and attenuation of irradiation within the flows were considered, and the absorbed irradiation at different depths was calculated based on the Beer-Lambert law. The model predictions for the steady state surface temperature of the inclined granular exposed to non-uniform radiative heat fluxes were strongly correlated to the experimental results with Pearson’s correlation coefficients >0.9. The analysis involved examining how particle temperatures varied over time at different depths within the flow. The flow velocities were observed to decrease with increasing depth, the lower part of the flow required more time to achieve steady-state conditions. At steady state, significant temperature differences between the surface and the bottom of the flow were observed, resulting from the low effective thermal conductivity of the granular flow. This finding showed that major heat transport occurs at the upper section of the flow.

These findings are crucial for designing solar receivers and reactors. Many studies^20^^,^^25^^,^^27^ assume that granular flow thicknesses are sufficiently thin to disregard variations in velocity and temperature, simplifying calculations to a single flow temperature using basic heat and mass balance. However, this study showed that even thin granular flows exhibit significant temperature gradients with respect to depth due to the non-Newtonian “fluid” characteristics, irradiation attenuation, and high thermal resistance. Relying on a simplified 1D heat and mass balance potential leads to substantial discrepancies in predicted versus actual flow temperatures, with particle temperatures higher than expected. The results further show that thermal transport was primarily driven by the top layer heat transfer, while bottom layers move more slowly at a lower temperature. While the total energy transported by the granular flow may be comparable in both simplified and detailed analyses, unexpectedly high particle temperatures potentially lead to critical system failures by exceeding operational temperature limits and increased radiative emissions to the surroundings. The fabrication, maintenance, and operational costs are substantial for high-temperature systems, and mechanical failures potentially prevent the system from achieving target economic performance, resulting in costly shutdowns. Additionally, elevated particle temperatures accelerate degradation of the particle thermophysical and optical properties, reducing receiver efficiency and energy storage performance over time.

### Limitations of the study

This work presents a comprehensive experimental investigation of granular flow behaviors along an inclined plane when directly heated by high-flux radiation. Despite significant effort in the experimentation, the characterization was limited to free-surface flow measurements. Understanding the flow properties and temperature distribution within the flow is crucial for understanding thermal transport in rapidly heated granular flows. A simple heat and mass transfer model was developed to predict heat transfer and transport mechanisms in the flow, providing unique insights into temperature distribution and heat transfer within the granular flow. However, the model has limitations in predicting energy transport due to the lack of experimental validation for particle velocity and temperature distribution within the flow. Simplifications in the model, such as a two-dimensional simulation domain, an empirical viscosity model, and an assumption of uniform particle volume fraction, potentially results in differences from the actual case. Nevertheless, the model still offers valuable insights into the heat transfer and transport mechanisms of particle flows exposed to high-flux radiation. The spatial and temporal flow and temperature results obtained from the experiment and the developed model can be expanded to a three-dimensional model, forming the foundation for analyzing the performance of particle solar receivers. More advanced computational modeling (e.g., MFiX or FLUENT) is available for solving the complex non-Newtonian “fluid” behavior with heat transfer by implementing previously measured temperature-dependent particle friction properties.^27^^,^^28^^,^^29^^,^^42^ This study focuses on a specific flow geometry of an inclined plane but further research on granular flow behaviors and heat transfer under rapid heating can be extended to free-falling particles and stair-step flows, which are widely employed for particle solar receiver applications. Ultimately, this study provides crucial insights into temperature-dependent granular flow behaviors and related heat transfer, essential for optimizing and developing efficient particle-based solar receivers and reactors. The knowledge gained can guide the design and operation of large-scale CSP systems, leading to improved solar-to-electric efficiency and more reliable performance estimations, which translates to lower levelized electricity costs. This research lays the groundwork for future advancements in the field, contributing to the development of more effective and sustainable solar energy solutions.

## Resource availability

### Lead contact

Further information and requests for resources should be directed to and will fulfilled by the lead contact, Professor Peter G. Loutzenhiser (peter.loutzenhiser@me.gatech.edu).

### Materials availability

This study did not generate new materials.

### Data and code availability

• All data reported in this paper will be shared by the lead contact upon request.
• No original code was used.
• Any additional information required to reanalyze the data reported in this paper is available from the lead contact upon request.

## Acknowledgments

This material is based upon work supported by the U.S. Department of Energy’s Office of Energy Efficiency and Renewable Energy (EERE) under Solar Energy Technologies Office (SETO) Agreement Number EE0008372. The particles used for this work were generously provided by CARBO.

## Author contributions

Conceptualization, S.Y.J. and P.G.L.; methodology, S.Y.J.; software, S.Y.J.; investigation, S.Y.J.; writing – original draft, S.Y.J.; writing – review and editing, S.Y.J., D.R., Z.Z., and P.G.L.; visualization, S.Y.J.; supervision, D.R., Z.Z., and P.G.L.; project administration, D.R., Z.Z., and P.G.L.; funding acquisition, D.R., Z.Z., and P.G.L.

## Declaration of interests

The authors declare no competing interests.

## STAR★Methods

### Key resources table

**Table:** 

REAGENT or RESOURCE	SOURCE	IDENTIFIER
**Software and algorithms**		

MATLAB	MathWorks	https://www.mathworks.com

**Other**		

Ceramic media	Carbo	https://carbo.tech/industries/energy/ceramic-media-forrenewable-energy/
High-density rigid alumina board	Zircar zirconia	https://www.zircarzirconia.com/images/datasheets/ZZ-5200_Rev03_-_Buster.pdf
High-purity alumina coating	Aremco	https://www.aremco.com/high-temp-refractory-coatings/

### Method details

#### Experimental procedures

The effects of non-uniform radiative heat fluxes on an inclined granular flow were investigated using a HFSS.^40^ The HFSS consists of seven 6 kW_e_ xenon short-arc lamps (Osram XBO 6000 W/HSLA) with a close match to the solar spectrum. The lamps are mounted in truncated ellipsoidal reflectors that share a common focus and provide an intense source of radiative heat. A cavity-type calorimeter was used to calibrate the incident intensity from the lamps, creating a near blackbody environment and minimizing spectral dependencies. The incident radiative heat fluxes from the HFSS were determined by considering heat absorbed by the cooling coils and other heat losses (*i.e.*, re-radiative and convective heat transfer from the cavity). A Lambertian target placed in the focal plane and CCD camera mounted behind the HFSS was used to capture images, and the pixel intensities were calibrated with calorimetry to estimate the spatial changes in radiative heat flux using the same lamp power settings.

An experimental apparatus for inclined granular flow was fabricated with the inclined plane strategically positioned at the focal plane of the HFSS (Figure 1). The inclined plane was fabricated from high-density rigid alumina board (Buster M-35, Zircar Zirconia) and spray coated with high-purity alumina coating (PyroPaint 634-AL, Aremco) to reduce surface erosion and ensure repeatability.^16^^,^^29^ The plane was inclined at 27° from horizontal, which was previously optimized to ensure steady, unaccelerated flows^29^ that enables steady granular flows. The radiative heat fluxes from the lamps were directly focused on the inclined plane (Figure 1) and free-surface velocities and temperatures of the granular flows were measured in this region. The dimensions of the experimental region were a length of *L* = 200 mm and a width of *W* = 80 mm. Sintered bauxite particles (Carbobead CP 30/60, CARBO) with effective diameters of *d*_p_ = 418 ± 59 μm^27^ were fed onto the inclined plane from a hopper. These particles underwent rapid heating within the irradiated region and were subsequently collected by the particle collector positioned at the end of the inclined plane. Alumina boards were placed on both sides of the inclined plane to keep the particles flowing along the inclined plane. An outer casing was fabricated using stainless steel to rigidly hold the inclined plane, and a particle storage bin was fabricated to provide granular flows for longer than 5 min and was placed at the top of the inclined plane. An outlet gate controlled by a servo motor was also fabricated to control the granular flow remotely. Support for the IR cameras and a high-speed camera were installed to fully capture the free-surface temperature and velocity fields of the heated particles. Mass flow rates were measured with temporal mass measurements from a force transducer attached to the particle collector. The particle bed height was measured using the laser displacement technique.^27^ A line laser was projected onto the inclined plane and vertical to the floor and a shift in projected line was observed as a function of particle bed thickness. The laser was projected onto the granular flow right below the exposed area due to difficulty in observing the laser within the heated region due to the HFSS. The shift in laser projection was previously calibrated using a stair-step feature with known step heights.^27^

A high-speed camera (Photron SA3) with a frame speed of 500 frames/s was used to measure free-surface velocity fields on the inclined plane granular flow coupled to PIV. The camera captured an *L*= 200 mm and a *W* = 80 mm of the plane where the high flux radiation was focused. Contrast improvement of the original image was achieved by employing a combination of a CLAHE technique and high-pass filters with window sizes of 20 and 15 pixels.^43^ Particle velocities were then calculated by analyzing consecutive images captured 12 ms apart. To compute the displacement of particles between these images, a direct Fourier transform was applied utilizing multiple passes of interrogation. Three passes were conducted with window sizes of 64, 32, and 16 pixels, respectively, and a step-size of 50%.^43^ Surface velocity vectors were determined based on these computations, and the velocity fields were measured using a calibration procedure involving a known-dimension checkerboard.^29^

IR cameras were used to measure free-surface particle temperature fields. The FLIR A655sc camera operates in the long wavelength IR (LWIR) range of 7.5-14 μm and was employed for temperature measurements ranging from 400 to 950 K. The FLIR A6261 camera equipped with an InGaAs detector operated in the short wavelength IR (SWIR) range of 0.9-1.7 μm for temperature measurements between 950 and 1500 K. The LWIR camera was calibrated with a hot-pressed Carbobead CP plate with surface temperatures measured by K-type thermocouples.^29^ To ensure that only the emitted thermal radiation from the surface of the granular flow was captured by the IR cameras, the penetration depth of radiation within the particles was analyzed. The results showed that the calculated penetration depth was smaller than the diameter of the particles within the spectral ranges of the IR camera measurements,^29^ indicative of the thermal emissions from particles beneath the surface absorbed within the underlying layers of the granular bed. The measurements obtained from the IR cameras, therefore, accurately represented the free-surface temperature of the granular flow. The SWIR camera was calibrated to filter out reflected radiation from the surface of the granular flow at shorter wavelengths outlined further in Supplement A. Three different average normal radiative heat fluxes from the lamp with $\bar{q}$″_HFSS_ = 400, 500, and 600 kW/m^2^ were used. The LWIR camera was able to provide temperature measurement of the heated granular flows <950 K with no influence of the reflected radiation and the SWIR provided overlapping temperature measurement <950 K.

#### Heat and mass transfer model

A pseudo, two-dimensional, continuum, non-steady heat and mass transfer model was developed to capture relevant transport mechanisms of gravity-driven granular flows along the inclined plane exposed to non-uniform radiative heat flux. A rectangular two-dimensional simulation domain (*L* × *δ*_bed_) situated in the *YZ* plane was defined within the granular flow region exposed to high flux radiation as shown in Figure 5. The domain was located at the center of the granular flow across the width of the inclined plane (in *X* direction) where the projected radiative heat flux on the flow is the strongest. Radiative heat flux on the flow and flow properties were assumed to be symmetric at the center of the flow for the model simplification. The simulation domain was discretized in both *Y* and *Z* directions. The governing energy equation for the granular flow is given as:

(Equation 2) $$\frac{\partial T_{p}}{\partial t}=-u_{p}\frac{\partial T_{p}}{\partial Z}+\alpha \left(\frac{\partial^{2}T_{p}}{\partial Z^{2}}+\frac{\partial^{2}T_{p}}{\partial Y^{2}}\right)+\frac{{\dot{q}}_{\text{abs}}}{{\left(\rho c_{p}\right)}_{\text{eff}}}$$

where thermal diffusivity is given as:

(Equation 3) $$\alpha =\frac{k_{p,\text{eff}}}{{\left(\rho c_{p}\right)}_{\text{eff}}}$$

and effective volumetric heat capacity of the flow is given as:

(Equation 4) $${\left(\rho c_{p}\right)}_{\text{eff}}=\varphi \rho_{p}c_{p,p}+\left(1-\varphi \right)\rho_{f}c_{p,f}$$

where *T*_p_ is the particle flow temperature; *t* is time; *u*_p_ is the particle flow velocity; $\dot{q}$_abs_ is the absorbed power density of irradiation from HFSS attenuates while penetrating through the flows; *k*_p,eff_ is the effective thermal conductivity of the particle flow; *φ* is the particle volume fraction of the flow; *ρ*_p_ is the solid particle density; *c*_p,p_ is the specific heat capacity of the solid particle; *ρ*_f_ is the fluid density; and *c*_p,f_ is the specific heat capacity of the fluid. The initial condition for *T*_p_ is given as:

(Equation 5) $$T_{p}\left(Y,Z,t=0\right)=300\;K$$

The boundary conditions at *Y* = 0 and *δ*_bed_ and *Z* = 0 for *T*_p_ are given, respectively, as:

(Equation 6) $$-k_{p,\text{eff}}{\frac{\partial T_{p}}{\partial Y}|}_{Y=0}=q_{\text{conv}}^{\prime \prime }+q_{\text{rad}}^{\prime \prime }$$

(Equation 7) $${\frac{\partial T_{p}}{\partial Y}|}_{Y=\delta_{\text{bed}}}=0$$

(Equation 8) $${T_{p}|}_{Z=0}=300\;K$$

where *q*′_conv_ is the convective flux from the flow surface to the ambient air; and *q′*_rad_ is the radiative heat flux from the flow surface to the surrounding environment. The thermophysical properties of the particles are listed in Table 3.^6^^,^^9^^,^^13^^,^^15^^,^^27^ At the uppermost layer of the granular flow (*Y* = 0), heat fluxes at the boundary were considered, including heat losses via *q*′_conv_ and *q*′_rad_. At the bottommost layer of the granular flow (*Y* = *δ*_bed_), an adiabatic boundary condition was adopted that considered the effective heat transfer reduction from the heated granular flow by the alumina insulating board. At the inlet of the granular flow (*Z* = 0), *T*_p_ = 300 K as the particles were continuously provided at room temperature. At the outflow boundary (*Z* = *L*), an open boundary condition was considered to ensure smooth outflows of the temperature field, maintaining continuity and preventing artificial reflections.

When granular flow was exposed to high radiative heat flux, the top particle layers do not absorb all irradiation. The radiative penetration within the flows was resolved with Beer-Lambert law. The radiation intensity decreased exponentially with a substantial portion of the irradiation absorbed by the top layer. However, due to the highly concentrated irradiation, the irradiation penetrating deeper into the flow was not negligible. The $\dot{q}$_abs_ in *Y* direction was derived from Beer-Lambert law, given as:

(Equation 9) $${\dot{q}}_{\text{abs}}=\kappa I_{0}e^{-\frac{\kappa Y}{\cos\;\theta }}$$

where *I*_0_ is the initial intensity of the radiation; *θ* is the angle of radiation respect to *Y*; and the absorption coefficient is calculated as:

(Equation 10) $$\kappa =Q_{\text{abs}}n_{p}A_{p}$$

where *Q*_abs_ is the absorption efficiency factor of the particle and Carbobead CP 30/60 was reported at 0.9; *n*_p_ is the particle number density of the flow; and *A*_p_ is the cross-section area of the particle. The incident angle of the radiation to the flow was assumed to be at 63° due to the inclined surface. This resulted in ∼80% of the irradiation absorbed by the first particle layer of the flow.

The effective thermal conductivity in the granular flow was predicted from Zehner, Bauer, and Schlünder (ZBS) model,^44^^,^^45^ given as:

(Equation 11) $$\frac{k_{p,\text{eff}}}{k_{f}}=\left(1-\varphi^{1/2}\right)\left(1-\varphi \right)\left[{\left(\frac{1}{\lambda_{G}}-\varphi \right)}^{-1}+\lambda_{r}\right]+\varphi^{1/2}\left[\varphi \lambda +\left(1-\varphi \right)\lambda_{c}\right]$$

where *k*_f_ is the thermal conductivity of the fluid; *λ*_G_ is the gas conduction ratio in Knudsen regime; *λ*_r_ is the radiative conduction ratio; *λ* is the solid-gas conduction ratio; *φ* is the empirical contact fraction of the solid particles where *φ* = 0.0031^41^; and *λ*_c_ is the relative thermal conduction ratio. The *λ*_G_ is given as:

(Equation 12) $$\lambda_{G}={\left(1+\frac{l_{m}}{d_{p}}\right)}^{-1}$$

where *l*_m_ is the modified mean free path of the gas molecules^44^; and *d*_p_ is the particle diameter with *λ*_r_ is given as:

(Equation 13) $$\lambda_{r}=\frac{\sigma }{\left(\frac{2}{\varepsilon_{p}}-1\right)}T_{p}^{3}\frac{d_{p}}{k_{f}}$$

where *σ* is Stefan-Boltzmann constant; and *ε*_p_ is the emittance of the particle. The *λ* is given as:

(Equation 14) $$\lambda =\frac{k_{p}}{k_{f}}$$

where *k*_p_ is the thermal conductivity of the solid particle, and *λ*_c_ is given as:

(Equation 15) $$\lambda_{c}=\frac{2}{N}\left\{\begin{array}{c} \frac{B\left(\lambda +\lambda_{r}-1\right)}{N^{2}\lambda_{G}\lambda }\ln\frac{\lambda +\lambda_{r}}{B\left[\lambda_{G}+\left(1-\lambda_{G}\right)\left(\lambda +\lambda_{r}\right)\right]}\\ +\frac{B+1}{2B}\left[\frac{\lambda_{r}}{\lambda_{G}}-B\left(1+\frac{1-\lambda_{G}}{\lambda_{G}}\lambda_{r}\right)-\frac{B-1}{N\lambda_{G}}\right] \end{array}\right\}$$

where *B* is the deformation parameter given as:

(Equation 16) $$B=1.364{\left(\frac{\varphi }{1-\varphi }\right)}^{1.055}$$

and *N* is a lumped term to simplify the relationship by combining terms, given as:

(Equation 17) $$N=\frac{1}{\lambda_{G}}\left(1+\frac{\lambda_{r}-B\lambda_{G}}{\lambda }\right)-B\left(\frac{1}{\lambda_{G}}-1\right)\left(1+\frac{\lambda_{r}}{\lambda }\right)$$

The convective heat transfer to the surroundings is given by Newton’s law of cooling as:

(Equation 18) $$q_{\text{conv}}^{\prime \prime }=h_{p-\text{air}}\left(T_{p}-T_{\text{air}}\right)$$

where *h*_p-air_ is the convective heat transfer coefficient between the granular flow surface and the ambient air. Local *h*_p-air_ in *Z* direction on the granular flow surface is given as^46^:

(Equation 19) hZ,p-air=0.332kfReZ1/2Pr1/3Z

where Re_*Z*_ is the local Reynold’s number; Pr is the Prandtl number; and *Z* is the location on the granular flow surface in *Z* direction. The *q″*_rad_ is given as:

(Equation 20) $$q_{\text{rad}}^{\prime \prime }=\sigma \varepsilon_{p}\left(T_{p}^{4}-T_{\text{air}}^{4}\right)$$

where *T*_amb_ is the measured surrounding temperature.

The *u*_p_ profile in the *Y* direction was determined from a viscosity model derived from previous experimental works for granular flows on an inclined plane. The test was performed at *d*_p_ ∼100 - 200 μm. The *u*_p_/*u*_p,s_ followed a function of (*δ*_bed_ – *Y*)/*δ*_bed_ with a parabolic dependence given as^47^:

(Equation 21) $$u_{p}=u_{p,s}{\left(1-\frac{Y}{\delta_{\text{bed}}}\right)}^{2}$$

The mass flow rate of the granular flow is calculated as:

(Equation 22) $$\dot{m}=\rho_{p}\varphi \int_{0}^{\delta_{\text{bed}}}u_{p}dY$$

where *φ* was assumed to be constant in the *Y* direction. The experimental observations indicated that the dimensionless surface velocity was proportional to the dimensionless thickness and independent of mass flow rate, given as:

(Equation 23) $$\frac{u_{p,s}}{\sqrt{gd_{p}}}=c\frac{\delta_{\text{bed}}}{d_{p}}$$

where *c* is the correlation coefficient and *g* is the local gravitational acceleration. Using this equation the velocity profile is given as:

(Equation 24) $$u_{p}=u_{p,s}{\left(1-\frac{c}{u_{p,s}}\sqrt{\frac{g}{d_{p}}}Y\right)}^{2}$$

Once the *u*_p,s_ is obtained, the *u*_p_ profile in the flowing layer was predicted. The *u*_p,s_ as a function of $\dot{m}$ from substitution is given as:

(Equation 25) $$u_{p,s}=\beta \sqrt{\dot{m}}$$

where

(Equation 26) $$\beta ={\left(3c\right)}^{1/2}{\left(\rho_{p}\varphi \right)}^{-1/2}{\left(3c\right)}^{1/4}$$

The resulting *u*_p_ profile is given as:

(Equation 27) $$u_{p}=\beta \sqrt{\dot{m}}{\left(1-\frac{c}{\beta \sqrt{\dot{m}d_{p}}}\right)}^{2}$$

The force balance along the flow between gravitation force and total shear stress for steady flows is given as:

(Equation 28) $$\rho_{p}\varphi gY\;\sin\;\theta +\eta \dot{\gamma }=0$$

where *η* is the apparent viscosity of the granular flow and $\dot{\gamma }$ is the shear rate of the granular flow. The proposed *η* is given as:

(Equation 29) $$\eta =-\rho_{p}\varphi g\delta_{b\text{ed}}\left(\frac{1}{\dot{\gamma }}+\frac{1}{2c}\sqrt{\frac{d_{p}}{g}}\right)\sin\;\theta$$

The shear rate $\dot{\gamma }$ is the derivative of *u*_p_ with respect to *Y*, given as:

(Equation 30) $$\dot{\gamma }=\frac{du_{p}}{dY}=-\frac{2u_{p,s}}{\delta_{b\text{ed}}}\left(1-\frac{Y}{\delta_{b\text{ed}}}\right)$$

The viscosity model showed that *η* depends on the material properties of *ρ*_p_ and *d*_p_, and flow parameters of *δ*_bed_, *φ* and *ϑ*, and local $\dot{\gamma }$. The flow-dependent characteristics of the granular flow are similar to Prandtl mixing-length theory for turbulent flows. Further derivation of the model and validation with a powder rheometer is available.^47^ This model was used to calculate up profile in the flow with the experimentally obtained *u*_p,s_ and *δ*_bed_.

The solution to the coupled partial differential equations was obtained using a method-of-lines approach, which involves imposing a spatial discretization to transform the model into a system of coupled ordinary differential equations. The model was implemented into MATLAB with the internal ordinary differential equation solver ODE15s to integrate the ordinary differential equations over time. A finite differencing method was employed for spatial discretization in the flow direction and over the bed height. A first-order upwind scheme and a central difference method were adopted for the first and second derivative terms, respectively, and a system of differential equation was obtained for each grid in the granular flow.

### Quantification and statistical analysis

We report the statistical analysis between experimental data and simulated results adopting Pearson’s correlation coefficient to provide a quantitative measure of linear relationships.
