# Supplementary material for: Exploring irradiated granular flows with rapid heating for concentrated solar thermal energy collection and storage
Source: iScience. 2025 Mar 4;28(4):112164. doi: 10.1016/j.isci.2025.112164 (PMC11982492; doi:10.1016/j.isci.2025.112164)
Supplement: Document S1. Figures S1 [file mmc1.pdf]

iScience, Volume *28*

Supplemental information

Exploring irradiated granular flows with rapid heating for concentrated solar thermal energy collection and storage

Shin Young Jeong, Devesh Ranjan, Zhuomin M. Zhang, and Peter G. Loutzenhiser

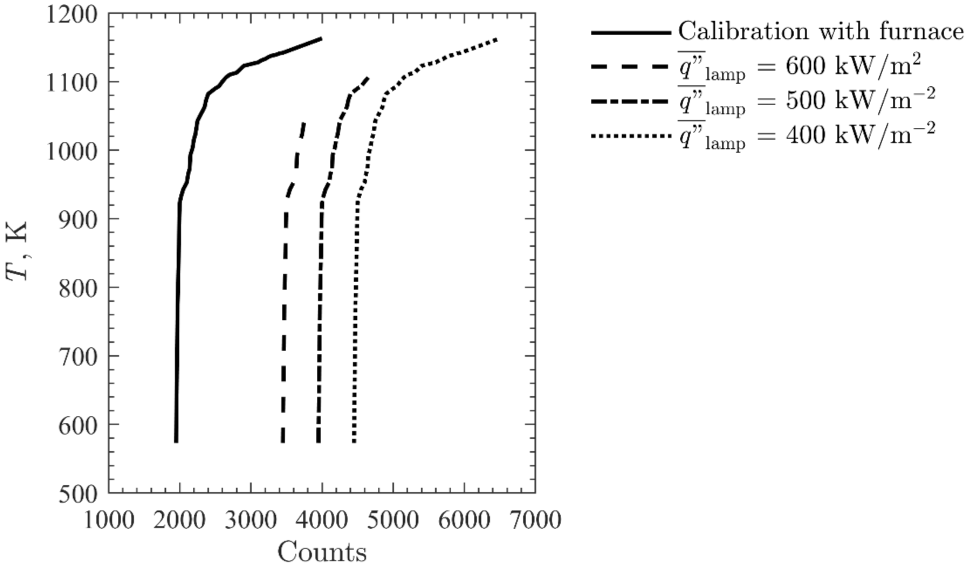

Figure S1. IR camera calibration for temperatures versus counts for FLIR A6261 with a neutral density filter between 573 K to 1273 K using a Carbobead CP plate with air furnace (solid line), and for the inclined granular flows exposed at average normal lamp radiative heat fluxes of 400 kW/m² (dotted line), 500 kW/m² (dashed-dotted line), and 600 kW/m² (dotted line). The SWIR camera calibration for the reflected radiation from the granular flow was performed by utilizing both SWIR and LWIR cameras. High heat flux from lamps were provided on the inclined plane granular flow and both cameras performed temperature readings. Three different normal radiative heat fluxes of $\bar{q}''_{HFSS}$ = 400, 500, and 600 kW/m² were used. The LWIR camera was able to provide temperature measurement of the heated granular flow below the temperature limit of the camera at 950 K with no influence of the reflected radiation. The SWIR camera also provided camera readings < 950 K and showed a very constant but increased value due to the influence of reflected radiation as shown in Figure A1. The measurements were performed for all three different lamp powers. The difference between the camera readings below 950 K using the heated Carbobead CP plate and irradiated granular flows showed the detection of reflected radiation from the granular flows. The increases in the SWIR camera readings with increase in the lamp power were obvious due to enhanced reflected radiation. The difference in the SWIR camera reading was used to subtract the SWIR camera readings for the granular flow at temperature > 950 K and the particle temperatures were determined using the calibrated results by the CP plate. The SWIR camera measurements on the heated granular flows along the inclined plane were performed above 950 K for each lamp power, and subtraction of counts accounting for reflected radiation was conducted. In Figure S1, the analyzed readings from the SWIR camera above 950 K were connected to the corresponding temperature. The maximum temperature of the granular flows was different for each lamp power.
